# Optimizing Mycobacterial Culture in Smear-Negative, Human Immunodeficiency Virus-Infected Tuberculosis Cases

**DOI:** 10.1371/journal.pone.0141851

**Published:** 2015-11-06

**Authors:** N. A. Ismail, H. M. Said, Z. Pinini, S. V. Omar, N. Beyers, P. Naidoo

**Affiliations:** 1 Department of Medical Microbiology, Faculty of Health Science, University of Pretoria, Pretoria, South Africa; 2 Centre for Tuberculosis, National Institute for Communicable Diseases, Johannesburg, South Africa; 3 Department of Medical Microbiology, University of Free State, Bloemfontein, South Africa; 4 TB/HIV Directorate, Gauteng Department of Health, Johannesburg, South Africa; 5 Desmond Tutu TB Centre, Department of Paediatrics and Child Health, Stellenbosch University, Cape Town, South Africa; University of Malaya, MALAYSIA

## Abstract

**Introduction:**

Tuberculosis (TB) is a significant public health problem and the diagnosis in human immunodeficiency virus (HIV)—infected individuals is challenging. The use of mycobacterial culture remains an important complementary tool and optimizing it has important benefits. We sought to determine the effect of an increase in the number of specimens evaluated, addition of nutritional supplementation to the culture medium, sputum appearance and volume on diagnostic yield and time to detection of pulmonary TB among smear-negative, HIV-infected adults.

**Methods:**

In this prospective study conducted at the Tshwane District Hospital and Academic TB Laboratory, Pretoria, South Africa we collected three sputum specimens an hour apart from presumptive TB cases at an antiretroviral treatment site. We analysed specimens from 236 patients. Specimen appearance and volume were recorded. All specimens were processed for culture using both standard and supplemented media.

**Results:**

A single specimen identified 79% of PTB cases using standard media; the second and third specimens added 12.5% and 8.3% respectively. Media supplementation, sputum appearance and specimen volume had no effect on culture yield or contamination rates. The mean time to detection was reduced from 19.8 days in standard cultures to 11.8 days in nutrient supplemented cultures (p = 0.002). For every 1 ml increase in sputum volume, time to detection was decreased by a factor of 0.797 (p = 0.011).

**Conclusion:**

Use of an inexpensive culture supplement substantially reduced time to detection and could contribute to reducing treatment delay among HIV-infected cases.

## Introduction

Tuberculosis (TB) remains a global health problem and is a leading cause of death in South Africa [1]. Human immunodeficiency virus (HIV) infection is an important contributory factor, increasing the risk for developing TB and making the diagnosis of TB more difficult [2–4]. Microbiological testing is important in the diagnosis of TB in HIV-infected individuals [5] as chest radiography and symptom screening have poor specificity[6, 7]. Whilst direct smear microscopy is widely used as the primary test for TB, its sensitivity in HIV-infected individuals is low, ranging from 31% to 69%[8]. The implication is that half of all TB cases may be missed when using microscopy alone for making the diagnosis of TB in HIV-infected individuals. This contributes to diagnostic delay which is associated with poorer outcomes, including death, especially among HIV-infected individuals [9, 10].

The introduction of the Xpert^®^ MTB/RIF (Xpert) assay is likely to reduce this problem as it has a higher sensitivity (79%) in HIV-infected individuals[11]. However this implies that where Xpert is used it could still miss 1 in 5 culture-positive cases. Additionally, the gap between the test availability globally and the diagnostic need is still large [12, 13]. Thus, better laboratory tests or improvements on existing technologies are required.

Mycobacterial culture, though more labour intensive, is more sensitive than both smear microscopy and Xpert and has been recommended for use in the diagnosis of TB in HIV-infected individuals including for smear- and Xpert-negative cases[14, 15]. The factors influencing the diagnostic yield of smear microscopy have been reviewed [16] but limited information is available on factors influencing the diagnostic yield of mycobacterial culture especially amongst HIV-infected individuals. Furthermore the potential diagnostic yield from second and third specimens with sputum culture is poorly studied and this information is needed especially amongst HIV-infected individuals where paucibacilliary disease is common. As culture is labour and cost intensive it is also important to assess the yields of culture from poor quality or low volume specimens to ensure this diagnostic tool is used more efficiently. The use of nutritional supplementation of culture is an alternative strategy which holds promise and requires further investigation.

This study aimed to determine the effect of an increase in the number of specimens evaluated, the addition of nutritional supplementation to the culture medium, sputum quality and volume on the diagnostic yield of culture and on time to detection of TB amongst smear-negative, HIV-infected cases.

## Materials and Methods

### Participants

In this prospective cohort study we recruited presumptive pulmonary TB (PTB) cases from the Comprehensive HIV and AIDS Care Centre at Tshwane District Hospital in Pretoria, South Africa, one of the main out-patient antiretroviral treatment initiation sites in the city.

We identified cases with at least three of the following symptoms: cough of any duration, fever of any duration, drenching night sweats or loss of weight. Only HIV-infected cases, above 18 years of age, not on TB treatment and with two smear-negative results in the last four weeks were included.

Patients in whom at least one of the three specimens collected was of insufficient volume (<0.1ml) for smear and culture were excluded. If any one of the three specimens collected was found to be smear-positive, the patient was also excluded from the study.

### Sample size

A sample size of 241 cases was determined to have 90% power using the logistic regression test (one sided) to detect a β of 0.887 (an odds ratio of 2.429) assuming a normally distributed covariate with correlation of covariates in the model at 0.5 and that the proportion of successes at the mean of 0.15. This sample size was increased to account for losses and for exclusion of cases with a positive smear in at least one of their three samples. A target sample size of 330 cases was chosen.

### Specimen and Data Collection

HIV-infected adults presenting to the TB focal point of the anti-retroviral treatment initiation clinic at Tshwane District Hospital were sequentially enrolled by the research assistant in the study after informed, written consent was obtained. A brief case report form (CRF) with unique study identifier, demographic and treatment history was completed by the research assistant through patient interviews and review of the clinical record. Each patient was instructed by the health care worker on how to produce sputum and was asked to provide three spot specimens at least an hour apart. The first specimen was collected at entry into the TB focal point after health care worker consultation, the second specimen was collected at least one hour later and the last specimen was collected prior to leaving clinic. Pre-barcoded laboratory request forms were completed for each specimen taken. Duplicate bar code labels from the laboratory form were used to link the laboratory request form to the specimen container and to the CRF, where it was placed adjacent to the relative collection time point (1,2 or 3) for each patient.

### Specimen Processing and Culture

All specimens were evaluated at the Tshwane Academic Department’s TB laboratory, a tertiary TB referral laboratory which provides services to the tertiary, provincial and district hospitals and the surrounding primary health clinics.

Sputum specimens collected at the clinical site were stored in a cooler box with ice packs and hand delivered to the laboratory at the end of the day by the research assistant. Specimens were refrigerated at 2–8°C overnight and processed the next morning. The laboratory research assistant was blinded to the order of the three specimens as specimens only had a barcode label and no other identifiers. The specimen’s appearance was assessed by visual inspection and graded as mucoid or purulent and then weighed using a domestic digital scale (Richter, South Africa) after correcting for the weight of the specimen container. Based on prior estimates, we took 1g of sputum as the equivalent of 1ml of sputum.

The specimens were digested and decontaminated using the NALC sodium hydroxide method [17] and centrifuged. Auramine smear microscopy testing was performed on the concentrated sediment to confirm that the specimen was smear-negative and preceded culture inoculation. The sediments from each specimen were cultured using BACTEC MGIT 960 (Becton Dickinson, USA) according to laboratory protocols. Two MGIT culture tubes were inoculated from each sample—one standard culture and one with an added nutritional enrichment supplement. The nutritional supplement (Bacto TB nutrient broth) was prepared as previously described by Brittle *et al*.[18]. The solution was stored in the fridge and replaced with a new batch in the latter part of the study.

The inoculation order was randomised to limit bias. This was achieved using the randomization function on a calculator: specimens with values generated between 0 and 0.5 (inclusive) had the standard culture tube inoculated first and those with values between 0.5 and 1 had the supplemented culture tube inoculated first. Positive cultures with acid fast bacilli observed on Ziehl Neelson staining were confirmed as *Mycobacterium tubercuolosis* complex (MTB) using the TBcID antigen test (Becton Dickson, USA) and those negative with this test were recorded as non-tuberculous mycobacteria (NTM).

Sputum quality, weight, smear result, culture result and time to detection (TTD) were recorded on the pre-barcoded laboratory report form for each specimen. At the end of the study the principal investigator linked the CRF and laboratory reports using the specimen barcode labels.

### Specimen Analysis

The proportion of specimens with a positive TB culture, with NTM and those contaminated were calculated using the total specimens tested in standard and supplemented media as the denominator. TB yield was calculated using the number of patients with at least one of three specimens with a positive TB culture as the numerator and the number of patients tested as the denominator. Incremental yield from the second specimen was calculated from the number of additional cases diagnosed from the second specimen that were not diagnosed from the first, divided by the total number of TB cases diagnosed. Incremental yield from the third specimen was the number of additional cases diagnosed from the third specimen that were not diagnosed by the first and second, divided by the total number of TB cases diagnosed. This analysis was repeated for supplemented media. The TTD was determined using the on-board MGIT instrument reporting system, from the loading of the tube into the instrument until growth was detected. Yield and TTD were stratified by specimen appearance, volume and type of culture medium.

### Data management

The CRF and laboratory forms were manually completed by the research assistants and securely stored with only the principal investigator having access to both documents. The data on these forms were entered onto an excel spread sheet using the CRF unique study number and associated laboratory bar codes for the analysis. Data were imported into STATA for analysis.

### Statistical Analysis

Specimen volume was not normally distributed and median data are presented. Time to detection was normally distributed and means are presented. We used logistic regression analysis, adjusted for clustering and paired data, to determine the effect of media type, specimen appearance and volume on TB yield. An accelerated failure time (AFT) model was used to assess their effect on TTD of mycobacterial growth. The AFT model is similar to the Cox proportional hazards model but regresses the logarithm of the survival time over the covariates, producing a more intuitive interpretation of survival analysis. For example, a coefficient of 0.5 indicates a reduction of survival time by this factor, meaning the event is experienced twice as fast.

### Ethical considerations

Ethics approval was obtained from the Ethics Committee of the Faculty of Health Science, University Pretoria and the Ethics Advisory Group of the International Union against Tuberculosis & Lung Disease. Permission to undertake the study was also obtained from the Gauteng Provincial Research Committee.

## Results

During January to December 2011 a total of 963 specimens were collected from 321 patients. Of these, 225 specimens were excluded as they were from patients in whom at least one of the three specimens collected was of insufficient volume (<0.1ml) for smear and culture and 30 were excluded because one or more of the patient’s specimens were smear-positive. Thus, a total of 708 sputum specimens from 236 patients were analysed. The mean age in the study population was 37 years (SD = 9 years), 61% were female and the mean CD4 count was 253 cells/mm^3^ (SD = 176 cells/mm^3^; n = 174).

MTB culture yield in standard media was 7.3% and in supplemented media 7.8% (uncorrected data; Table 1). The incremental TB culture yield for the second and third culture in standard media was 12.5% and 8.3% respectively, while in supplemented media this was 7.7% and 11.5%. The odds of a positive culture in supplemented media was 1.145 times (95% CI 0.439 to 2.984; p = 0.782) that of standard media (Table 2). Rates of contamination were not significantly different between standard media (12 specimens, 1.7%) and supplemented media (6 specimens, 0.8%; p = 0.285).

**Table 1:** Culture positivity for *Mycobacterium tuberculosis*, incremental yield and time to positivity in standard and supplemented MGIT cultures.

**MGIT culture with standard media**				
	Specimen 1 (n = 236)	Specimen 2 (n = 236)	Specimen 3 (n = 236)	Total (n = 708)
Number and % culture positive*	19 (8.1%)	15 (6.4%)	18 (7.6%)	52 (7.3%)
Incremental number new TB positive#	19	+3	+2	24
Incremental yield new TB positive#	79.2% (19/24)	+12.5% (3/24)	+8.3% (2/24)	100%
Mean time to detection (days) (95% CI)	18.5 (16.0 to 21.1)	20.5 (16.3 to 24.8)	20.4 (17.1 to 23.8)	19.8 (18.0 to 21.6)
Number NTM (%)	2 (0.8%)	3 (1.3%)	0 (0%)	5 (0.6%)
Number contaminated (%)	4 (1.7%)	5 (2.1%)	3 (1.3%)	12 (1.7%)
Median volume (variance)	1.7 (3.4)	1.55 (1.6)	1.6 (1.9)	1.65 (2.3)
**MGIT culture with supplemented media**				
	Specimen 1 (n = 236)	Specimen 2 (n = 236)	Specimen 3 (n = 236)	Total (n = 708)
Number and % culture positive*	21 (8.9%)	18 (7.6%)	16 (6.8%)	55 (7.8%)
Incremental number new TB positive#	21	2	3	26
Incremental yield new TB positive#	80.8% (21/26)	7.7% (2/26)	11.5% (3/26)	100%
Mean time to detection (days) (95% CI)	12.1 (10.5 to 13.8)	12.1 (10.2 to 14.0)	11.1 (9.4 to 12.7)	11.8 (10.9 to 12.8)
Number NTM (%)	3 (1.3%)	2 (0.8%)	1 (0.4%)	6 (0.8%)
Number contaminated (%)	2 (0.8%)	2 (0.8%)	2 (0.8%)	6 (0.8%)

*Each specimen analysed independently.

^#^Sequential specimens adding only new sputum-positive TB not identified in the previous sample(s).

Table shows unadjusted yield data for the three specimens taken from each HIV-infected, smear-negative, presumptive TB case and grown in both standard and supplemented media. Abbreviations: TB = tuberculosis; MGIT = Mycobacterial Growth Inhibitor Tube; CI = confidence interval; NTM = non-tuberculous mycobacteria.

**Table 2:** The effect of media type, specimen appearance and volume on TB yield.

	Odds Ratio	Robust Standard Error	p-value	95% Confidence Interval
Supplemented media	1.145	0.560	0.782	0.439 to 2.984
Appearance—purulent	2.179	2.344	0.469	0.265 to 17.939
Volume	1.132	0.139	0.311	0.890 to 1.439
Constant	0.008	0.004	0.000	0.004 to 0.020

The table shows the logistic regression analysis, adjusted for clustering and paired data, to determine the effect of media type, specimen appearance and volume on TB yield.

The majority of specimens were mucoid (639/708 or 90.3%;Table 2). There was no significant difference in TB yield between mucoid and purulent specimens (OR 2.179; 95% CI: 0.264 to 17.939; p = 0.469). The median overall specimen volume was 1.65 ml (variance 2.29) and specimen volume had no effect on TB yield.

**Fig 1:**
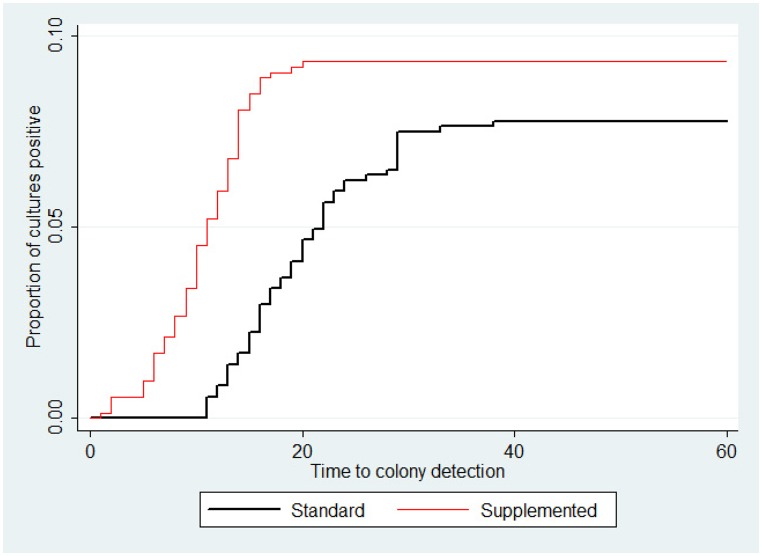
Proportion of cultures positive by time in days of standard and supplemented cultures.

The TTD was reduced from a mean of 19.8 days (95% CI: 18.0 to 21.6 days) with standard media to a mean of 11.8 days (95% CI: 10.9 to 12.8 days) with supplemented media. The accelerated failure model showed that TTD in supplemented media was reduced significantly by a factor of 0.630 (95% CI: 0.472 to 0.840; p = 0.002) compared to standard media (Table 3, Fig 1). Sputum appearance did not have a significant effect, with TTD in purulent samples reduced by a factor of 0.377 compared to mucoid samples (95% CI: 0.047 to 3.031, p = 0.359). However, for every 1 ml increase in specimen volume, TTD decreased by a factor of 0.797 (95% CI: 0.670 to 0.950; p = 0.011) which was significant.

**Table 3:** The effect of media type, specimen appearance and volume on mycobacterial growth time to detection.

	Time Ratio	Robust Standard Error	p-value	95% Confidence Interval
Supplemented media	0.630	0.093	0.002	0.472 to 0.840
Appearance—purulent	0.377	0.401	0.359	0.047 to 3.031
Volume	0.797	0.071	0.011	0.670 to 0.950
Constant	6559	4108	0.000	1921 to 22 391

This table shows outputs from an accelerated failure model used to assess the effect of media type, specimen appearance and volume on mycobacterial growth time to detection (after adjustment for clustering and paired data)

## Discussion

There is an urgent need to improve the diagnosis of TB, amongst HIV-infected patients who are smear-negative. Despite the introduction of Xpert in South Africa, liquid culture remains an important complementary tool to ensure active cases of TB not detected by rapid diagnostics are identified and treated. However, costs associated with liquid cultures are often a limiting factor in resource limited settings and identifying determinants that increase the likelihood of a positive culture are important for effective application of this technology.

The first collected sputum diagnosed 79% of TB cases with standard MGIT medium showing that a single sputum culture detects the majority of these cases. The addition of a second and third sputum specimen for culture increased the yield by 12.5% and 8.3%, respectively. This is similar to observations in Thailand among HIV-infected patients which showed that the first specimen yielded 71% of positive results, the second specimen an additional 17% with the third specimen an additional 10% [19]. However the Thailand study also included specimens from lymph node biopsies to improve the diagnosis of TB. Thus, submitting additional specimens for liquid culture may be required for clinically relevant cases as a single culture does not definitively rule out TB in HIV-infected individuals.

No difference in yield was observed between standard and supplemented media. This differs from a study by Brittle et al. [18] that used the same nutritional supplementation in paediatric specimens. The reason for this difference is unknown. It may be related to different strain types with specific growth requirements that may not be present in our area. Alternately, the use of gastric aspirates in the paediatric population may have driven the nutritional requirements for growth in the harsh gastric medium.

We showed that TTD in liquid culture was significantly reduced by a factor of 0.630 with the addition of a simple and cheap (< $0.01/sample) nutritional supplement (from a mean of 19.8 to 11.8 days). The study by Brittle et al. had similar findings with mean TTD reduced from 18.5 to 12.4 days [18]. The major limitation of culture is its slow turnaround time and the findings from the current and previous study [18] using a specific nutrient supplement provide evidence which could significantly advance this technology among paucibacillary TB patients, where it is needed most.

The influence of specimen appearance on yield of TB showed no difference between those that were mucoid or purulent. Specimen volume also did not affect yield, although we observed faster TTD with higher volumes of sample. Thus attention to specimen volume at time of collection is important. We have not evaluated pooling of specimens as an alternate approach but this should be an area of future research which could reduce costs substantially. Other studies have shown a positive relationship between bacillary load and TTD and is possibly due to higher volumes of sample leading to the higher bacillary load and thus shorter TTD [20]. It should be noted that only 73% of patients (236/321) were able to produce 3 specimens of sufficient volume for testing which is a known difficulty in diagnosing TB in this sub-set of presumptive TB cases [21, 22].

### Limitations

Patients were at an advanced stage of HIV disease (mean CD4+ count 253 cells/mm^3^) and we may therefore have under-estimated the effects on yield and TTD compared to those with less immunosuppression. We only included patients who could provide three consecutive specimens of at least 0.1 ml and may have excluded those cases with the lowest bacillary counts, resulting in our study over-estimating yield. Additionally, due to the short time frame between specimens collected, yields may have been lower than expected. Lastly, we did not assess the influence of ART or CD4+ counts on TB culture yield.

## Conclusion

This study has shown that an inexpensive supplement to liquid MGIT cultures substantially reduced the time to culture detection without increased culture contamination and with no decline in yield. A single culture identified most cases of pulmonary TB but additional specimens may be required to exclude the diagnosis in HIV-infected, smear-negative cases. The cost-effectiveness of analysing additional specimens needs to be assessed.

## Supporting Information

S1 Dataset Sputum volume and weight data for each patient. “Culture” = specimen culture result without supplementation; “ID” = mycobacterial identification of culture without supplementation; “TTD” = time to detection (in days) for culture without supplementation; “Culture_SUP” = specimen culture result with supplementation; “ID_SUP” = mycobacterial identification of culture with supplementation; “TTD_SUP” = time to detection (in days) of culture with supplementation; Gender: M = Male F = Female; MTB = *Mycobacterium tuberculosis;* NTM = non-tuberculosis mycobacterium.
(XLS)
